# Evaluation of the anti-feeding and insecticidal effects of a topically administered combination of imidacloprid and permethrin (Advantix®) against *Phlebotomus* (*Larroussius*) *perniciosus* (Newstead, 1911) in dogs following monthly administration

**DOI:** 10.1186/s13071-018-2690-2

**Published:** 2018-03-02

**Authors:** Emilie Bouhsira, Katrin Deuster, Emmanuel Lienard, Christophe Le Sueur, Michel Franc

**Affiliations:** 1 Ecole Nationale Vétérinaire de Toulouse, 31076 Toulouse Cedex, France; 2 Bayer Animal Health GmbH, 51368 Leverkusen, Germany; 3 Bayer Animal Health, 92254 La Garenne Colombes, France

## Abstract

**Background:**

Two laboratory experiments (Studies 1 and 2) were conducted to confirm the efficacy of an imidacloprid and permethrin combination (Advantix® Spot-on, Bayer) to repel and kill *Phlebotomus* (*Larroussius*) *perniciosus* sand flies when applied once a month topically to dogs.

**Methods:**

Both studies compared dogs treated with a combination containing 100 mg/ml imidacloprid + 500 mg/ml permethrin (Advantix® Spot-on, Bayer) to placebo treated dogs. The treatments were applied topically on Day -28 (Study 2) and Day 0 (Studies 1 and 2). Sand fly exposures with 80 unfed females were performed before the first treatment for allocation purposes and post-treatment on study days (SDs) 1, 7, 14, 21 and 28 (following first or second monthly treatment for Studies 1 and 2, respectively). After 60 min, sand flies were assessed for mortality and engorgement status.

**Results:**

Repellent evaluation (anti-feeding effect) on all days post-infestation showed efficacies that ranged between 88.1–99.3% during the first month and 92.2–98.9% during the second. Analyses of the comparison of fed sand fly counts for each treatment group resulted in a highly significant reduction (*P* < 0.0001) at all post-infestation time points for those dogs treated with Advantix®. A significant (*P* < 0.0001 for all time points) insecticidal effect was equally demonstrated. No treatment related adverse events were observed during the study.

**Conclusions:**

In the present studies Advantix® Spot-on demonstrated to be safe and to provide excellent four-week sand fly (*P. perniciosus*) repellency of ≥88.1% and ≥92.2% after a first and second monthly treatment, respectively. A significant insecticidal effect was also observed.

## Background

A significant number of phlebotomine sand fly species are present in Europe, and their geographical distribution is dependent on temperatures with a highest relative probability of the presence of *Phlebotomus perniciosus* in areas where daytime summer temperature range between 25 °C and 33 °C, whereas the lower limit temperature is predicted at 16.5 °C [1]. Changes in distribution have been observed over the past decades and species have been captured in more northerly countries and at higher altitudes [2–10]. *Phlebotomus* (*Larroussius*) *perniciosus* (Diptera: Psychodidae) is known to be currently present in Bulgaria, Croatia, France, Macedonia, Malta, Portugal, Spain, southern Switzerland and western Germany and has recently been found for the first time in Andorra [8, 10]; its geographical reach increased in Italy [11, 12], and is predicted to expand in Spain and Germany due to favourable climate changes [13, 14].

In south-west Europe and north-west Africa, the bloodsucking females of *P. perniciosus* are the most widespread sand fly vectors of *Leishmania infantum* (Kinetoplastida: Trypanosomatidae), the parasitic protozoan that causes most visceral and cutaneous leishmaniosis in humans and canine reservoir hosts in the region [15–18]. Female sand flies do not seem to have a clear host preference but rather opportunistically feed on hosts to which they have the easiest access. It is therefore likely that in urban and peri-urban settings humans and domestic dogs are the main targets for sand flies [19]. Other sand fly-borne infections, i.e. phleboviruses causing diseases in humans ranging from short self-limiting fevers to encephalitis and fatal haemorrhagic fever, have been reported in Europe for the last decades [20, 21]. Further spread is feared because underlying phenomena persist: movements of vectors and animals, and environmental changes notably related to global warming [10, 22, 23].

Prevention of sand fly bites in dogs can be achieved by using effective topical veterinary products that exert repellent effects (e.g. permethrin-based) [24–27]. The additional insecticidal effect of repellent products used on dogs reduces the risk of human infection with *L. infantum* [28–30]. The objective of the present studies was to confirm the repellent and insecticidal efficacy of a combination containing 100 mg/ml imidacloprid + 500 mg/ml permethrin (Advantix® Spot-on, Bayer) against sand flies (*P. perniciosus*) on dogs following a first and second monthly treatment.

## Methods

Two laboratory studies were conducted at the National Veterinary School of Toulouse, France. The agent used in the studies is a common sand fly in the Mediterranean Basin and one of the main vectors of canine leishmaniosis. No alternative to an *in vivo* evaluation is available.

### Animals

Fourteen healthy adult Beagle dogs were used in each study (Study 1: 10 females, 2 males and 2 spayed males, weighing 8.31 kg to 12.90 kg at inclusion; Study 2: 11 females, 1 neutered female and 2 spayed males, weighing 8.63 kg to 11.14 kg at inclusion). They had not been exposed to short-acting ectoparasiticides for 3 months before inclusion in this work and had never been treated with any long-acting ectoparasiticides. The dogs were housed in accordance with the European animal welfare regulations in individual indoor boxes (approximately 4 m^2^ per dog) in a controlled environment with approximately 12 h light and 12 h darkness. Each dog was identified with the number of a subcutaneously implanted microchip. They were fed with a commercial dry dog food in an appropriate ration and had water available *ad libitum*. Dogs were maintained and handled with due regard for their well-being and were acclimatized to the environment for two weeks before the first treatment. They were observed daily for their general health condition throughout the respective study and remained healthy all along the animal phase.

### Sand fly exposures

The sand fly strain used in this study originated from Lisbon, Portugal, and had been maintained under laboratory conditions for 10 years without being exposed to any insecticide. The sand fly females are fed on rabbit blood, and the larvae are maintained on specific medium containing rabbit faeces. The cycle from egg to adult lasts for 5 to 7 weeks, at optimal conditions of ambient temperature (25–30 °C) and relative humidity (70–80%).

Sand fly exposure was induced using laboratory reared adults (unfed females only). All animals were infested with *P. perniciosus* females for a total of six times. Twenty-four hours before each infestation, female sand flies were aspirated from their breeding cage with a vacuum pump and were placed in sand fly proof nets (80 per net) with access to sugary-water-soaked cotton. They were fasted approximately 2 h before exposure to dogs by removing the cotton from the cages. The first infestation was conducted within two weeks before the first treatment and was used for allocation purposes only. Five post-treatment infestations were conducted on days 1, 7, 14, 21 and 28 after the first and second treatment for Studies 1 and 2, respectively. On each infestation day, animals were weighed and sedated by intramuscular injections of a mixture of dexmedetomidine (Dexdomitor®, Elanco Santé Animale, Lilly, Suresnes, France) and ketamine (Clorketam®, Laboratoire Vetoquinol S.A., Lure, France). Once the effects of anesthesia were visible, they received an intramuscular injection of diazepam (Valium®, Roche injectable, Neuilly s/ Seine, France) at a dose rate of 4 μg/kg, 9 mg/kg and 5 mg/dog, respectively; The dogs were then placed in individual infestation proof nets containing sand flies. When needed, re-dosing with a combination of dexmedetomidine and ketamine by IM injection was performed. Once the first dog was introduced in its infestation net, the light was dimmed. During infestation, treated and control dogs were placed in separated infestation rooms where temperature and relative humidity were maintained at 18 °C and 27 °C and between 40 and 60%, respectively.

After 60 min of exposure, dogs were carefully taken out of the net and examined for any dead sand flies (engorged or unengorged), and then returned to their box. At the end of the exposure period, all live and dead sand flies were collected, counted and recorded as unengorged or engorged. The engorgement status was determined by visual observation at the naked eye of distension of the abdomen and the presence of blood.

After each sand fly challenge, the nets were thoroughly cleaned.

### Allocation and treatment

Each study followed a randomised block design based on the number of engorged (dead + live) *P. perniciosus* sand flies collected from each dog after the pre-treatment infestation. Dogs meeting the inclusion criteria were allocated to one of the study groups (7 dogs per group) after the first infestation and before the first treatment. Dogs were ranked according to the number of engorged (dead + live) sand flies in descending order. Animal ID number was used to break ties. The dogs were then introduced into blocks of two animals. Dogs were randomly allocated into 2 groups by drawing lots. Study 1: Group 1 dosed with the recommended pipette size (Advantix® Spot-on for dogs over 4 kg up to 10 kg or Advantix® Spot-on for dogs over 10 kg up to 25 kg) delivering ≥10 mg/kg body weight (BW) imidacloprid and ≥50 mg/kg BW permethrin. This dose corresponded to ≥0.1 ml Advantix® Spot-on per kg BW and Group 2 placebo treated. Treatment was performed on Day 0. Study 2: Group 1 dosed with the recommended minimum therapeutic dose (10 mg/kg BW imidacloprid and 50 mg/kg BW permethrin). This dose corresponded to 0.1 ml Advantix® Spot-on per kg BW and Group 2 placebo treated. Treatments were performed on Days -28 and 0. For dose calculation, the body weight of the dogs was rounded to two decimal places. For all treated animals in both studies, the formulation was applied according to manufacturer’s instructions by parting the hair and applying the formulation directly on the skin. Dogs weighing ≤10.0 kg were treated in one spot between the shoulder blades while dogs weighing > 10.0 kg up to and including 20 kg received the dose volume distributed evenly to four spots on top of the back, from the shoulder to the base of the tail. Care was taken not to spill any product. Dogs were restrained for about 1 min following administration to allow the product to spread. All dogs were observed at 2 and 4 h after treatment for any adverse reactions to the product.

### Data analyses

The data collected on each occasion were the numbers of live and engorged, live and unengorged, dead and engorged, and dead and unengorged sand flies. Effects were assessed on a group basis (the total number of each type of sand fly in each group of seven dogs). The statistical unit was the individual dog.

#### Repellent (anti-feeding) efficacy

Repellent efficacy of the combination was assessed by comparing the number of fed (live or dead) sand flies in the treated animals to the number of fed (live or dead) sand flies in the placebo treated control group for each infestation day. The repellent efficacy was determined as follows:

$$ \mathrm{Repellent}\ \mathrm{efficacy}\ \left(\%\right)=\left[\left(\mathrm{MFC}\hbox{--} \mathrm{MFT}\right)/\mathrm{MFC}\right]\times 100 $$

where MFC is the mean number of fed sand flies in control dogs, and MFT is the mean number of fed sand flies in treated dogs.

In addition to the classical approach of evaluating the product effect based on Abbott’s formula and providing a measure of percent repellency, the ratio of fed to unfed female sand flies at each time point was calculated for each study separately.

#### Insecticidal efficacy

Insecticidal efficacy (measured by knockdown effect after 60 min) of the combination was assessed by comparing the number of live (fed or unfed) sand flies in the treated animals to the number of live sand flies in the placebo treated control group for each infestation day (i.e. one hour after sand fly infestation).

The insecticidal efficacy (at one hour after infestation) was determined as follows:

$$ \mathrm{Insecticidal}\ \mathrm{efficacy}\ \left(\%\right)=\left[\left(\mathrm{MLC}\hbox{--} \mathrm{MLT}\right)/\mathrm{MLC}\right]\times 100 $$

where MLC is the mean number of live sand flies in control dogs, and MLT is the mean number of live sand flies in treated dogs.

#### Statistical methods

Descriptive statistics were generated for both study groups for each challenge time point after treatment. Both arithmetic and geometric mean counts were calculated for both types of sand fly counts (live and fed).

The two central tendencies (geometric means *vs* arithmetic means) for use in the efficacy calculations were evaluated by examining the distributions within each treatment group for each given time point count. Data with approximately normal distributions would indicate that the arithmetic means would be appropriate to use in the efficacy calculation, whereas non-normal distributions would suggest a transformation would be necessary, before calculating the percent efficacy.

## Results

No health abnormalities related to treatment were observed throughout the studies.

Both, the live and fed counts were found to be nearly normally distributed, and thus arithmetic means were used to calculate efficacy and an analysis of variance (ANOVA) parametric method using treatment group as the only fixed effect was applied to the actual sand fly counts (non-transformed) for each period post-infestation.

High numbers of sand flies that had fed were recovered from all dogs at the allocation infestation meaning that all dogs in both studies demonstrated an equal and high pre-treatment parasite holding ability. Then all control dogs maintained an adequate number of engorged females throughout the study (with means between 57.9 and 68.6 out of the 80 sand flies, Table 1). The mean number of sand flies found alive in the control group after the hour of challenge remained high all along the study: between 75.0 and 77.4 sand flies per control dog, supporting the robustness of the challenge model.

**Table 1:** Percent repellency of *Phlebotomus perniciosus* on dogs treated with the combination of imidacloprid and permethrin based on arithmetic means

Exposure day	Number of engorged sand flies				Repellency (%)	
	Control dogs		Treated dogs			
	Study 1	Study 2	Study 1	Study 2	Study 1	Study 2
1	58.6	57.9	0.4	2.0	99.3**	96.5**
7	62.0	62.6	1.9	0.7	97.0**	98.9**
14	64.6	63.0	7.7	2.3	88.1**	96.4**
21	65.4	63.9	7.7	5.0	88.2**	92.2**
28	68.6	63.9	6.0	4.4	91.3**	93.1**

**Significant difference between the population means of the treated and control groups (*P* < 0.0001)

### Repellency (anti-feeding)

In the Advantix® treated groups average counts of sand flies that had fed ranged from 0.4 to 7.7 per time point, while average counts of sand flies that had fed ranged from 57.9 to 68.6 in the control animals. Treated dogs had significantly fewer fed sand flies at the end of the exposure period than placebo treated control dogs for all study days (ANOVA: *F*_(1,12)_ > 72, *P* < 0.0001 for all data analysed). The sand fly repellency after 60 min exposure was 99.3, 97.0, 88.1, 88.2 and 91.3% (Study 1) and 96.5, 98.9, 96.4, 92.2 and 93.1% (Study 2) for Days 1, 7, 14, 21 and 28, respectively (Table 1).

**Fig. 1:**
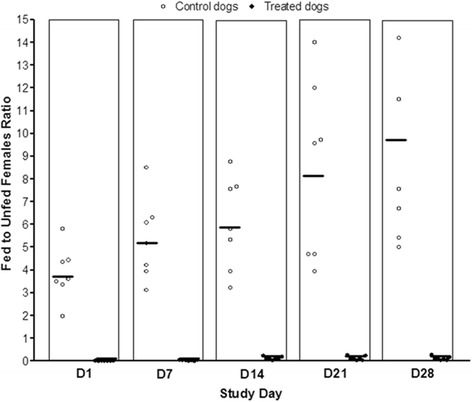
Fed to unfed females ratios Study 1. Mean of ratios (bars) from control dogs are shown on the left and those from treated dogs in the right of each column. One fed/unfed ratio value from an untreated control dog exceeding 15.0 is not shown, although it was included in the mean of ratios calculation (SD28 = 25.0)

**Fig. 2:**
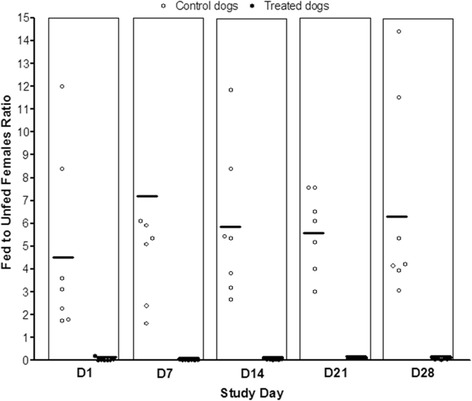
Fed to unfed females ratios Study 2. Mean of ratios (bars) from control dogs are shown on the left and those from treated dogs in the right of each column. One fed/unfed ratio value from an untreated control dog exceeding 15.0 is not shown, although it was included in the mean of ratios calculation (SD7 = 25.3)

The calculation of the fed to unfed female ratio showed high values and a wide distribution of feeding rates of *P. perniciosus* on control animals in contrast to homogeneous clusters of very low fed to unfed ratios among treated dogs at each time point for both studies (Figs. 1 and 2).

### Insecticidal effect

In the Advantix® treated group average counts of sand flies that survived exposure ranged from 0 to 36.3 per time point, while average counts of sand flies still alive at the end of the exposure ranged from 74.0 to 76.7 in the control animals. Treated dogs had significantly fewer live sand flies at the end of the exposure period than placebo treated control dogs for all study days in both studies (ANOVA: *F*_(1,12)_ > 86, *P* < 0.0001 for all data analysed). The insecticidal effect after 60 min exposure was 100, 100, 93.6, 95.0 and 79.1% (Study 1) and 76.8, 81.7, 73.0, 58.9 and 52.3% (Study 2) for Days 1, 7, 14, 21 and 28, respectively.

## Discussion

A commercial formulation containing imidacloprid and permethrin (Advantix® Spot-on, Bayer) applied at the minimum recommended label dose provided significant repellent (anti-feeding) efficacy against the sand fly *P. perniciosus* for four weeks following monthly administrations in dogs. The sustained anti-feeding efficacy of ≥88.1% and ≥92.2% was observed from 1 to 28 days post first and second monthly treatment, respectively. Miro [31] evaluated the activity of the same combination following a single topical treatment and reported the anti-feeding efficacy of > 92.7% for three weeks (based on arithmetic means) with efficacy slightly reduced below the 80% threshold on day 28. Anti-feeding efficacy over 80% is considered as a minimum threshold with levels preferably exceeding 90% by regulatory authorities [32]. The very low fed to unfed ratios observed in treated dogs in both studies clearly indicate a strong and sustained effect in preventing *P. perniciosus* from having a blood meal on dogs These laboratory findings are supported by field data obtained by Otranto et al*.* [29]. The results show that Advantix® Spot-on reduces the risk of *Leishmania* transmission under natural conditions in endemic areas significantly. A reduction of 88.9% of *Leishmania infantum* infection was obtained in dogs treated every four weeks with Advantix® Spot-on with incidence density rates of infection significantly lower than in the untreated control group (*P* < 0.05).

These results, present and historical, confirm that regular monthly treatments throughout the sand fly activity season will provide continued high efficacy levels and thus help in the prevention of sand fly infestation and *Leishmania infantum* transmission in dogs. This level of efficacy was also observed in other similar studies testing topical combinations including permethrin [33, 34]. The short-term insecticidal efficacy (knockdown effect) was evaluated as a second parameter and was ≥93.6% for three weeks post treatment and dropped to 79.1% in the fourth week in Study 1. In Study 2 a less pronounced short-term insecticidal efficacy (≥ 73.0%) was observed for two weeks post treatment and dropped to levels below 60% afterwards. The knockdown effect reported here is consistent with the results observed with other permethrin-containing products in equivalent studies [35].

## Conclusions

The combination of imidacloprid and permethrin demonstrated a significant repellent effect against *P. perniciosus* bites on the dogs which lasted for four weeks after first treatment. The repellent efficacy was also demonstrated to last for four weeks after a second treatment. Therefore, the results suggest that in endemic areas, the application of this product every four weeks throughout the vector season would be a good tool to reduce sand fly bites significantly. It should, therefore, be included in an integrated leishmaniosis control program in dogs. The education of owners about arthropod-borne pathogens and the importance of such prevention programmes is paramount to increase compliance and is anticipated to become more important with the forecasted spread of competent vectors to currently non-endemic areas.
